# Identification of genomic variants putatively targeted by selection during dog domestication

**DOI:** 10.1186/s12862-015-0579-7

**Published:** 2016-01-12

**Authors:** Alex Cagan, Torsten Blass

**Affiliations:** Department of Evolutionary Genetics, Max Planck Institute for Evolutionary Anthropology, Deutscher Platz 6, 04103 Leipzig, Germany

**Keywords:** Genomics, Domestication, Artificial selection, Natural selection, Behavioural genomics

## Abstract

**Background:**

Dogs [*Canis lupus familiaris*] were the first animal species to be domesticated and continue to occupy an important place in human societies. Recent studies have begun to reveal when and where dog domestication occurred. While much progress has been made in identifying the genetic basis of phenotypic differences between dog breeds we still know relatively little about the genetic changes underlying the phenotypes that differentiate all dogs from their wild progenitors, wolves [*Canis lupus*]. In particular, dogs generally show reduced aggression and fear towards humans compared to wolves. Therefore, selection for tameness was likely a necessary prerequisite for dog domestication. With the increasing availability of whole-genome sequence data it is possible to try and directly identify the genetic variants contributing to the phenotypic differences between dogs and wolves.

**Results:**

We analyse the largest available database of genome-wide polymorphism data in a global sample of dogs 69 and wolves 7. We perform a scan to identify regions of the genome that are highly differentiated between dogs and wolves. We identify putatively functional genomic variants that are segregating or at high frequency [> = 0.75 Fst] for alternative alleles between dogs and wolves. A biological pathways analysis of the genes containing these variants suggests that there has been selection on the ‘adrenaline and noradrenaline biosynthesis pathway’, well known for its involvement in the fight-or-flight response. We identify 11 genes with putatively functional variants fixed for alternative alleles between dogs and wolves. The segregating variants in these genes are strong candidates for having been targets of selection during early dog domestication.

**Conclusions:**

We present the first genome-wide analysis of the different categories of putatively functional variants that are fixed or segregating at high frequency between a global sampling of dogs and wolves. We find evidence that selection has been strongest around non-synonymous variants. Strong selection in the initial stages of dog domestication appears to have occurred on multiple genes involved in the fight-or-flight response, particularly in the catecholamine synthesis pathway. Different alleles in some of these genes have been associated with behavioral differences between modern dog breeds, suggesting an important role for this pathway at multiple stages in the domestication process.

**Electronic supplementary material:**

The online version of this article (doi:10.1186/s12862-015-0579-7) contains supplementary material, which is available to authorized users.

## Background

Dogs [*Canis lupus familiaris*] are considered the first animal species to be domesticated by humans. Genetic and archaeological evidence suggests that this process began approximately 11-16kya [1, 2]. Dogs and their closest living relatives, wolves [*Canis lupus*] differ in a variety of phenotypic traits, despite only differing in ~0.04 7 % of nuclear coding-DNA sequence [3]. Particular attention has been given to their behavioral differences, with dogs showing a greater ability to read human communicative behaviour [4]. When and how these new cognitive abilities emerged remains unclear. It has been suggested that rather than selection for these specific behaviors it was selection for tameness, a reduction in fear and aggression towards humans, that permitted the expression of these latent abilities, which are inhibited in wolves by their fear response [5, 6].

Unlike the majority of domestic species, which were primarily selected for production related traits, dogs were typically selected for their behaviors [7]. Modern breeds are the result of human mediated selection for an incredibly wide-range of behaviors, including guarding, herding and pointing [8]. Pioneering early work on breed crosses demonstrated a genetic basis to some of these behavioral differences between breeds [9, 10]. Since then much work has been done to identify the genetic basis of phenotypic differences between dogs breeds. The great phenotypic diversity and population structure between modern dog breeds has proven to be a powerful model for elucidating the genetic basis of breed-specific traits [3]. Studies have utilized a variety of approaches included trait mapping [11, 12] selection scans [12, 13] and candidate gene driven approaches [14, 15].

There has been much success in identifying genetic variants underlying morphological traits, which often have a relatively simple mono-allelic genetic architecture [12, 16, 17]. Identifying the genetic basis of behavioral traits, which are typically assumed to have a more complex genetic architecture, has proven to be a more challenging endeavor [3]. Nevertheless, canine behavioral genetics is a rapidly moving field and several studies have made progress in uncovering the genetic variants associated with behavioral differences between breeds [8, 18, 19].

One behaviour of particular interest is aggression, given that selection for reduced aggression towards humans was likely a prerequisite for domestication [20]. Indeed, dogs generally show reduced fear and aggression towards humans compared to wolves [21]. Candidate gene approaches have identified significant allele frequency differences that correlate with levels of aggression related behaviour within or between dog breeds in genes that have previously been associated with aggression in humans. Examples include monoamine oxidase B [*MAOB*] [22], the dopamine D4 receptor [*DRD4*] [23], the dopamine transporter [*SLC6A3*] [24], tyrosine hydroxylase [*TH*] [25] and dopamine beta-hydroxylase [*DHB*] [25]. One study tested 62 SNPs occurring within or close to 16 neurotransmitter-related genes for allelic associations with aggression [26]. Although multiple risk or protective haplotypes for aggression were identified no single haplotype was in complete association with the phenotypes recorded, supporting the view that aggressive behaviour in dogs has a complex genetic basis. Taken together these results suggest that selection for behavioral traits related to aggression in dogs has targeted a variety of pathways, particularly those involving the synthesis, transport and degradation of neurotransmitters such as dopamine.

Despite this progress the genetic changes underlying reduced fear and aggression in dogs relative to wolves remain unknown. It is not necessarily the case that the genes associated with breed-specific behaviors are the same ones that were selected during the early domestication process. Indeed, despite the success of breed mapping approaches, their dependence on inter-breed variation makes them unsuitable to identify genetic variants selected for during the early domestication process that are shared by all dog breeds. While the findings of studies that focus on intra-breed variation may not be generalizable across breeds. As a result we know less about the genetic basis of the phenotypic changes that occurred during the early stages of dog domestication and differentiate all dogs from their wild progenitors than we do about differences between modern dog breeds.

Identifying the genetic changes that occurred early in the domestication process thus necessitates additional approaches beyond comparisons between breeds. Gene expression studies have identified sets of genes that are differentially expressed in the brains of dogs and wolves [27, 28] and between aggressive and non-aggressive dog breeds [29], however whether these contribute to behavioral differences remains unknown. Previous work using scans for selection in genomic data from dogs and wolves has identified genomic regions that may have been targeted by selection during early dog domestication [30–34]. In most cases the putative causative genomic variants underlying these selection signals remain to be identified. In most cases the putative causative genomic variants underlying these selection signals remain to be identified. One of the few cases where the causative variant has been identified is the gene *AMY2B*. Axelsson et al. [32] found that modern dogs have increased copy numbers of the pancreatic amylase gene *AMY2B* compared to wolves, potentially an adaptation to a starch rich diet associated with human co-habitation. Although a later study found that this variation is polymorphic and does not represent a truly fixed genetic difference between dogs and wolves [1].

Thus far the putatively functional variants that are fixed or segregating at high frequency between dogs and wolves have not been systematically characterized. One exception is the study of Li et al. [35], in which non-synonymous variants segregating for alternative alleles between dogs and wolves were identified. However, this study was limited by a relatively small sample size [three wolves and five dogs], meaning that many of the sites they identified may not be truly segregating between all dogs and wolves. Furthermore, they only considered non-synonymous variants as putatively functional. Identifying and studying the properties of a wide range of putatively functional variants is of interest because they are expected to include the alleles that were selected during dog domestication and are responsible for the phenotypic differences between dogs and wolves. Furthermore, studies that rely solely on selection scans to identify adaptive loci are liable to false positives due to hitchhiking of neutral variants, particularly in populations that have experienced strong bottlenecks [36], such as domestic dogs [1]. Prioritising candidate regions that contain putatively functional variants is one way to increase the likelihood of identifying the true selective sweeps.

We analyzed variants that are fixed or segregating at high frequency between dogs and wolves. We identified these variants using DoGSD, the largest available dataset of whole-genome polymorphism data from dogs and wolves [37]. Of these variants we identify a subset as being putatively functional. We combine this information with a genomic scan for selection to identify regions of the genome that are highly diverged between dogs and wolves. We perform Gene Ontology analysis of genes with putatively functional variants segregating at high frequency between dogs and wolves. We find that putatively functional changes influencing genes involved in adrenaline biosynthesis appear to have been particularly targeted by selection during dog domestication. We find that selection during dog domestication appears to have been strongest around variants influencing protein structure. Furthermore, we identify 11 genes with putatively functional variants that appear to be fixed for alternative alleles between dogs and wolves. These changes are of particular interest because they may be the genetic variants responsible for the phenotypic differences between all dogs and all wolves that may have been selected during dog domestication.

## Results and discussion

### Scan for selection

**Fig. 1:**
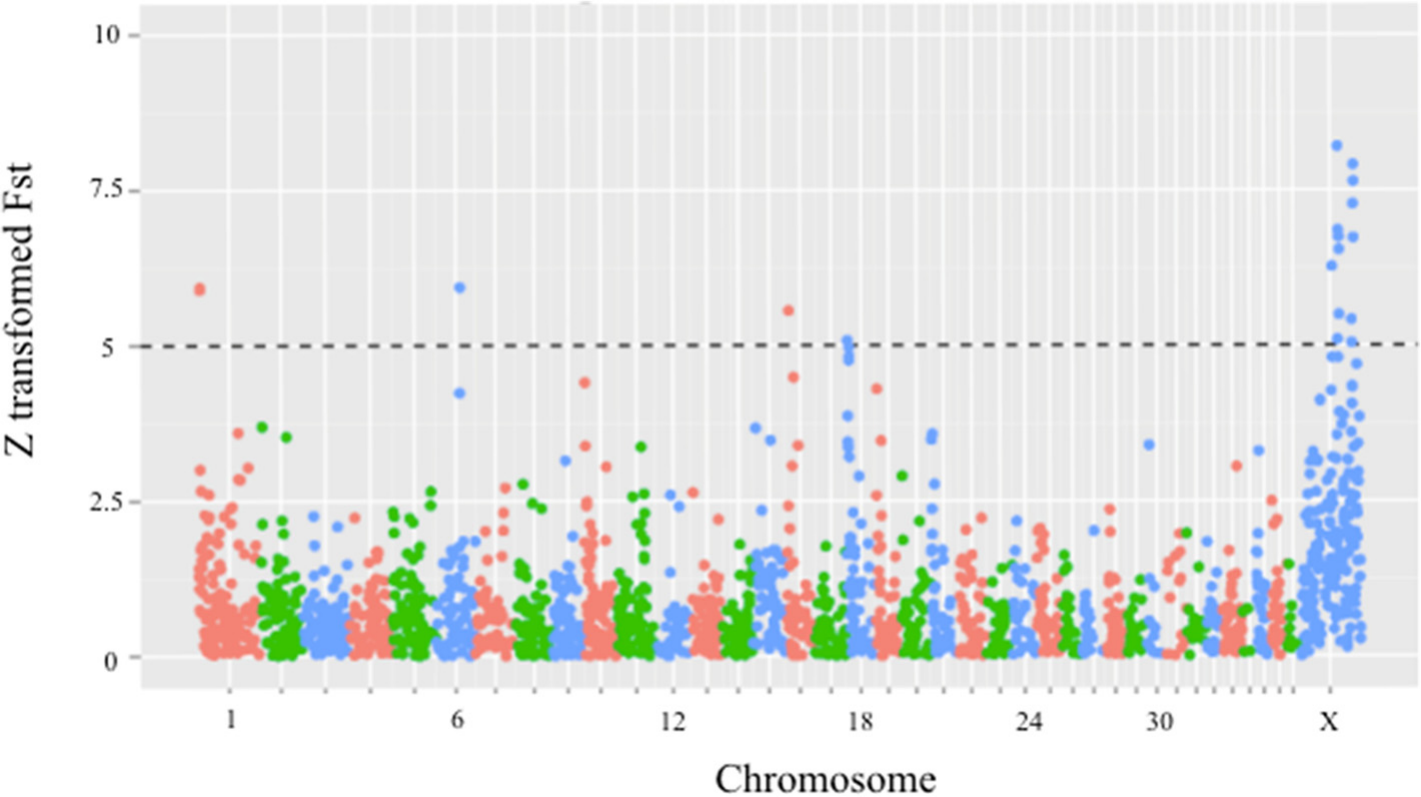
Genome-wide scan for selective sweeps. Z-transformed mean Fst calculated in 500kb genomic windows across the autosomes and X chromosome between dogs and wolves. Each point represents a 500kb window. A dashed horizontal line represents our threshold for identifying putatively selected regions (>5 Z(Fst)). 18 windows exceed this threshold and are considered as putative selective sweeps

To identify genomic regions that may contain variants that were selected during dog domestication we identified regions that were highly diverged between dogs and wolves by calculating the mean Fst between dogs and wolves in 500kb windows along the genome. Although previous studies have performed window-based scans for signatures of selection in dogs and wolves [30, 32], none have been performed on such a large sample of either species using whole-genome data. Following Axelsson et al. [32] we Z transform our Fst scores and consider regions scores that fall at least five standard deviations from the mean (Z(Fst)) as putatively selected (Fig. 1).

Mean levels of divergence are higher on the X chromosome (X chromosome mean Fst = 0.21 compared to 0.14 for autosomes). This is usually attributed to the smaller effective population size of the X chromosome due to its mode of transmission [38]. However, it is also possible that this signal is partially the result of artificial selection during domestication having occurred disproportionately on the X chromosome. As males are hemizygous for X-linked traits this may have provided humans with an opportunity to easily identify and select recessive alleles on the X chromosome. As the penetrance of any given genetic variant in a population is dependent on its allele frequency and its mode of dominance, regardless of underlying demographic history, we use the same threshold to identify putatively selected regions on the X chromosome and the autosomes. We acknowledge that this may result in a higher false positive rate on the X chromosome. When the X chromosome is considered independently no regions on the X chromosome fall over five standard deviations above the mean Fst score. Nevertheless, as mentioned above, the X chromosome may contain functional variation contributing to dog domestication and we do not want to miss true positives through an overly stringent cut-off. Therefore, following Li et al. [35], we include the X chromosome in our selection analyses.

Using these criteria we identify 18 regions with strong signatures of population divergence between dogs and wolves (Table 1). As expected from the higher levels of mean divergence on the X chromosome, 13 of these regions are on this chromosome. 14 of these 18 regions contain genes which are candidates for being targets of selection. We identify many regions previously found to be under selection in dogs, including a region on chromosome 1 containing *MBP*, which encodes myelin basic protein, and a region on chromosome 16 which contains *MGAM*, involved in starch metabolism [32].

**Table 1:** Genes in 500kb windows with Z transformed mean Fst scores five standard deviations above the mean

Chromosome	Window [bp]	Mean Fst	Genes in window
1	2500001–3000000	0.427497	SNORA70, GALR1 MBP, ZNF236
1	3000001–3500000	0.429086	U6, ZNF516
6	47000001–47500000	0.429446	RNPC3
16	7000001–7500000	0.411249	PRSS58, MGAM, TAS2R38, CLEC5A, PRSS37, U6, TAS2R5, TAS2R3, SSBP1, WEE2
18	500001–1000000	0.388113	
X	66000001–66500000	0.445258	ZNF711, POF1B, 7S
X	77000001–77500000	0.53831	TCEAL1, MORF4L2, GLRA4, TMEM31, PLP1, RAB9B, SLC25A53, 7SK,
X	77500001–78000000	0.388753	FAM199X, ESX1
X	78000001–78500000	0.473412	
X	79500001–80000000	0.46818	U6, TBG, MUM1L1
X	80000001–80500000	0.458387	U6, CXorf57, RNF128, RNF128, TBC1D8B, CLDN2, RIPPLY1, MORC4
X	80500001–81000000	0.407971	RBM41, NUP62CL, PIH1D3
X	105500001–106000000	0.404133	MOSPD1, ZNF75D, U6, ZNF449, DDX26B
X	106000001–106500000	0.386268	SLC9A6
X	108000001–108500000	0.493717	
X	108500001–109000000	0.524088	FGF13, cfa-mir-504
X	109000001–109500000	0.467526	
X	109500001–110000000	0.511206	F9, MCF2, U4, ATP11C, cfa-mir-505, CXorf66

The selection scan was performed on a larger and more geographically diverse dataset than previous scans for selection comparing dogs and wolves [30–35]. We note that while our dataset was chosen to sample as broadly as possible from the worldwide distribution of dog and wolf populations that our dog sample is particularly enriched for German Shepherds [11], Tibetan Mastiffs [11] and indigenous dogs [39]. Therefore, the sweep signals that we detect may be shared only among these breeds and not truly reflect universal signatures of selection across dog breeds. Future studies with sampling from across a wider range of breeds will be necessary to confirm whether these regions have elevated divergence between all dog and wolf populations.

To explore whether the elevated mean Fst in these regions could be explained by neutral evolutionary processes rather than selection we performed coalescent simulations for the autosomal genome based on a neutral model of the demographic history of these samples (Materials and Methods). We simulated 500kb haplotypes for all samples and calculated mean Fst between the pooled dog and wolf haplotypes so that the results could be compared to our empirical data. The mean of the mean Fst scores across all simulations is slightly elevated, Fst = 0.184, compared to the mean Fst of our real data, Fst = 0.144, or when excluding the X chromosome, Fst = 0.140. Despite this elevated mean Fst, we never observe simulated 500kb regions with mean Fst scores as high as our putatively selected regions (Additional file 1: Figure S3). The highest mean Fst score from the simulations is 0.31, while the lowest mean Fst score of the 18 putatively selected regions is 0.39. Therefore, the simulations suggest that the cut-off we use to detect putatively selected regions is conservative and the elevated mean Fst scores of these regions are unlikely to have been the result of purely neutral evolutionary forces.

### Variants fixed for alternative alleles between dogs and wolves

As many of these putatively selected regions contain multiple genes the identification of the targets of selection is challenging. There may also be selected variants that are not surrounded by the signatures of a selective sweep. This could occur for a variety of reasons, including when selection occurs on standing genetic variation [40] and because strong population bottlenecks reduce our ability to detect signatures of selection over neutrality [36]. Both these scenarios appear to have occurred during the process of dog domestication [1].

To try and identify the targets of selection in these putatively selected regions as well as selected variants not surrounded by signatures of selection we identified all single nucleotide positions that were fixed for alternative alleles between dogs and wolves (Fst = 1). From this list of 2112 sites we used Ensembl’s Variant Effect Predictor (VEP) to identify those which had putatively functional consequences [41] (Materials and Methods).

We identify only 11 genes with putatively functional positions that appear fixed for alternative alleles between dogs and wolves (Table 2). Eight of these fall within the selective sweep regions. Of the remaining four, three are in 500kb windows directly neighbouring the candidate selective sweep regions. The remaining gene, *RELT*, is in the ninth most highly diverged 500kb region between dogs and wolves on chromosome 21. Therefore, the majority of fixed putatively functional variants are found regions within highly diverged regions, suggesting that for dog domestication a hard sweep model may be appropriate for detecting selected variants. The relatively low Ne of the population ancestral to all dogs, estimated to be as low as 700–3,200 [1], combined with the high selection coefficients possible under artificial selection, may have increased the likelihood of hard sweeps relative to other non-domesticated species where selection has been studied, such as *Drosophila melanogaster* [42].

**Table 2:** Putatively functional variants fixed for alternative alleles between dogs and wolves

Gene ID	Gene name	Position [chr:position]	Nucleotide change [Dog/Wolf]	Predicted effect
*FGF13*	Fibroblast growth factor 13	X:108729524	C/G	5‘-UTR
*FHL1*	Four and a half LIM domains 1	X:106604107	A/G	3‘- UTR
*F9*	coagulation factor IX	X:109533147	C/A	3‘- UTR
*MAP7D3*	MAP7 domain containing	X:106609169	C/T	3‘- UTR
*MBP*	Myelin basic protein	1:2951693	G/C	3‘- UTR
*MCF2*	MCF.2 cell line derived transforming sequence	X:109544224	G/C	3‘- UTR
*RELT*	Relt tumor necrosis factor receptor	21:24836981	G/A	3‘- UTR
*RNPC3*	RNA-binding region containing 3	6:47026666	T/G	Missense [T/P]
*RNPC3*	RNA-binding region containing 3	6:47035497	A/C	Splice region, intronic
*SLC9A6*	Solute carrier family 9, subfamily A	X:106463600	T/C	3‘- UTR
Novel protein coding	ENSCAFG00000018988	X:108560105	T/C	Missense [I/T]
Novel protein coding	ENSCAFG00000018988	X:108560351	A/G	Missense [Q/R]
Novel protein coding	ENSCAFG00000018988	X:108560422	G/A	Missense [E/K]
Novel protein coding	ENSCAFG00000018988	X:108560629	A/G	Missense [M/V]
Novel protein coding	ENSCAFG00000023289	X:77456592	G/A	Missense [E/K]

A previous study on dog domestication by Li et al. [35] identified 26 non-synonymous variants that were fixed for alternative alleles between dogs and wolves. Using our larger dataset we were able to further refine this list. Of the 26 non-synonymous variants they identified, only six appear as true substitutions between dogs and wolves in our analysis. Five of these six substitutions fall in two genes of unknown function on chromosome X (*ENSCAF00000018988* and *ENSCAF00000023289*). The remaining substitution falls in *RNPC3* on chromosome 6.

### Fixed variants potentially contributing to behavioral differences

Three of the 11 genes with putatively functional variants fixed for alternative alleles between dogs and wolves are involved in brain development and may therefore potentially contribute to the behavioral differences between dogs and wolves. Of the six genes in the 1Mb candidate sweep region we detect on chromosome one only one gene has a putatively functional variant fixed between dogs and wolves. The gene, *MBP*, encodes myelin basic protein and the segregating site occurs in the 3′-UTR. Myelin basic protein is a component of the myelin sheath, which influences the velocity of axonal impulse conduction [43]. Socially isolated mice show deficits of myelination in the prefrontal cortex, suggesting that myelination is sensitive to behavioral changes [39]. Furthermore, children with autism are significantly more likely to produce anti-MBP antibodies than controls [44].

Intriguingly, another gene that is highly expressed in myelinated nerve fibers [45], *FGF13*, is fixed between dogs and wolves for a putatively functional segregating site in its 5′-UTR. *FGF13* encodes fibroblast growth factor 13 and is within the 500kb region with the second strongest signal of population divergence between dogs and wolves (Table 1). FGF13 is a growth factor involved in neuronal migration in the cerebral cortex during development [46]. Overexpression of *FGF13* in neuronal cultures from rat embryonic cortex increases the number of neurons containing gamma-aminobutyric acid (GABA) [47], which is notable for the important role of GABA in the regulation of behaviour, including fear [47] and aggression [48]. The presence of a fibroblast growth factor in our list of candidates is potentially supportive of the ‘domestication syndrome’ hypothesis, which predicts that many of the traits observed in domestic animals are the result of selection on genes related to embryonic development, including fibroblast growth factors [49]. Which of these phenotypes, if any, were targeted by selection will require further investigation.

Perhaps the most intriguing variant fixed between dogs and wolves occurs in the 3′-UTR of *SLC9A6*, which encodes sodium/hydrogen exchanger protein 6. This protein regulates the endoluminal pH of early and recycling endosomes involved in the trafficking of proteins essential for the plasticity of glutaminergec neurons [50]. Loss of function mutations in this gene in humans can lead to Christianson syndrome, also known as “Angelman-like syndrome” [51]. Phenotypes typical of patients with loss of function mutations in *SLC9A6* include cognitive developmental delays, absence of speech, stereotyped repetitive hand movements, hyperkinetic movements and postnatal microcephaly with a narrow face [51, 52]. Christianson syndrome is also frequently characterised by a happy disposition with easily provoked laughter and smiling, an open mouth with excessive drooling and frequent visual fixation on hands [51, 52]. Several of these phenotypes resemble those that distinguish dogs from wolves. Therefore it is tempting to speculate that selection on regulatory variation influencing expression of *SLC9A6* may have played an important role in producing some of the behavioral phenotypes that emerged during dog domestication.

### Variants potentially contributing to anatomical differences

Dogs and wolves are also anatomically distinct [53]. One gene we detect with a variant in the 3′-UTR fixed for alternative alleles between dogs and wolves is *FHL1*, which encodes Four and a half LIMB domains 1. *FHL1* is most highly expressed in skeletal muscle [54]. Defects in this gene in humans result in a variety of muscle disorders, for example scapuloperoneal myopathy, characterized by progressive weakening of shoulder and lower leg muscles [55, 56]. Selection on this gene may have contributed to the reduced efficiency of skeletal musculature that has been observed in dogs relative to wolves.

Another gene potentially contributing to morphological differences between dogs and wolves is *RNPC3*, which encodes the protein RNA-binding region containing 3. RNPC3 is involved in pre-mRNA U12-dependent splicing. *RNPC3* is one of only two genes with more than one putatively causal variants fixed between dogs and wolves, the other is a gene of unknown function (Table 2). One variant causes a non-synonymous change while the other is in a predicted intronic splice site. Notably, *RNPC3* is the only autosomal gene with a non-synonymous substitution segregating between all wolves and dogs. Mutations in this gene in humans cause pituitary related growth hormone deficiencies, potentially by disruption of the growth hormone pathway [57]. This pathway also involves the genes *IGF1* and *IGFR1*, both are associated with haplotypes influencing body size between dog breeds [16, 58], suggesting that this pathway may have been repeatedly targeted by selection for body size during dog domestication.

Interestingly, *RNPC3* is situated less than 1Mb from *AMY2B*, which it has been argued has been selected for increased copy number in dogs as an adaptation to a starch-rich diet [32]. The close proximity of these two genes suggests that the putatively functional variants in *RNPC3* may have risen as a result of hitchhiking, due to selection on the neighbouring *AMY2B*, or vice versa. It is an intriguing possibility that selection in dogs on AMY2B for dietary adaptations could have led to morphological changes through the hitchhiking of non-selected functional alleles in the neighbouring *RNPC3*. Further work will be necessary to untangle the original targets of selection in this case.

### Pathway enrichment suggests selection on behaviour

It is not necessarily the case that fixed phenotypic differences between populations must have a fixed genetic basis, particularly in the case of complex polygenic traits. Therefore, we also looked for variants that are not fixed between dogs and wolves. To do this we identified all single nucleotide positions that were highly differentiated between dogs and wolves (Fst > = 0.75). From this list of 199,821 sites we used VEP to identify those which had putatively functional consequences. We identify 848 genes with putatively functional variants showing an allele frequency difference of > = 75 % between dogs and wolves.

We performed a gene ontology enrichment analysis on these 848 genes using the gene ontology and analysis software PANTHER [59, 60]. The only pathway to show a significant enrichment is the ‘adrenaline and noradrenaline biosynthesis pathway’ (*P*-value = 4.19E-08) (Table 3). Given the key role of adrenaline in the fight-or-flight response [61] and noradrenaline’s key role as a hormone and neurotransmitter responsible for vigilant attention [62] it is possible that this is driven by genes that have been targeted by selection for changes in behaviour, such as tameness, during dog domestication.

**Table 3:** Panther pathways gene enrichment analysis of genes containing variants with an Fst score > = 0.75

PANTHER Pathways	Canis familiaris - REFLIST [19662]	Genes with putatively functional variants [848]	Expected number	Fold enrichment	*P*-value
Unclassified	17318	712	746.91	-	0.00E00
Adrenaline and noradrenaline biosynthesis	26	9	1.12	+	4.19E-08
Axon guidance mediated by netrin	38	7	1.64	+	2.31E-01
Dopamine receptor mediated signaling pathway	51	8	2.20	+	2.97E-01
Nicotine pharmacodynamics pathway	31	6	1.34	+	3.87E-01
Alpha adrenergic receptor signaling pathway	22	5	.95	+	4.45E-01
Gonadotropin releasing hormone receptor pathway	225	19	9.70	+	7.74E-01

The enrichment signal is the result of putatively functional variants in nine genes (Table 4), including the monoamine oxidases *MOAO* and *MAOB*. The proteins encoded by these genes are involved in the deamination of dopamine, serotonin, adrenaline and noradrenaline. In humans variants in *MAOA* have been associated with aggression [63]. Inhibition of MAOA and MAOB during brain development induces pathological aggressive behaviour in mice [64] and transgenic mice deficient for MAOA show aggressive behaviour and alterations in levels of noradrenaline in the brain [65]. Another gene we identify is *TH*, which encodes tyrosine hydroxylase, the rate-limiting enzyme in the synthesis of dopamine and noradrenaline [66]. Tyrosine hydroxylase catalyzes the conversion of L-Tyrosine into L-Dopa. Startlingly, the gene encoding DOPA decarboxylase (Aromatic-L-Amino-Acid decarboxylase), which transforms L-Dopa into dopamine, also has a putatively functional variant segregating at high frequency between dogs and wolves (Table 4). This gene, *DDC*, is also involved in several other decarboxylation reactions related to neurotransmitter synthesis, including the conversion of 5-HTP to serotonin [67]. Both *DDC* and *MAOB* have been associated with attention-deficit/hyperactivity disorder in humans [68]. We also detect putative functional variants segregating at high frequency in three genes which encode neurotransmitter transporters in the solute carrier 6 family (SLC6). Proteins in the SLC6 family are involved in the plasma membrane transport of dopamine, noradrenaline, serotonin and GABA and are involved in neurotransmitter signaling [69]. Overall these results strongly suggest that there has been selection for changes in neurotransmitter metabolism during dog domestication, particularly in the catecholamine biosynthesis and transport pathways, which include dopamine, adrenaline and noradrenaline.

**Table 4:** Putatively functional variants with an Fst score > = 0.75 in genes in the ‘adrenaline and noradrenaline biosynthesis’ pathway

Gene ID	Gene name	Position [chr:position]	Nucleotide change [Dog/Wolf]	Predicted effect
*DDC*	Dopa decarboxylase [Aromatic-L-Amino-Acid decarboxylase]	18:1806717	C/T	Missense [R/Q]
*MAOA*	Amine oxidase [flavin-containing] A	X:37747023	T/C	3‘-UTR
*MAOB*	Amine oxidase [flavin-containing] B	X:37766049	C/T	3‘-UTR
*SLC6A3*	Sodium-dependent dopamine transporter member 3	34:11239621	C/T	Splice region, intronic
*SLC6A17*	Sodium-dependent neutral amino acid transporter member 17	6:41776709	C/A	3‘-UTR
*SLC6A19*	Sodium-dependent neutral amino acid transporter member 19	34:11329939	G/A	3‘-UTR
*SNAP29*	Synaptosomal-associated protein 29	26:30614788	C/T	Missense [S/N]
*SNAP29*	Synaptosomal-associated protein 29	26:30607948	G/A	3‘-UTR
*SNAP29*	Synaptosomal-associated protein 29	26:30607975	T/C	3‘-UTR
*SNAP29*	Synaptosomal-associated protein 29	26:30608088	T/C	3‘-UTR
*SNAP29*	Synaptosomal-associated protein 29	26:30608209	G/A	3‘-UTR
*SNAP29*	Synaptosomal-associated protein 29	26:30608212	T/C	3‘-UTR
*SNAP29*	Synaptosomal-associated protein 29	26:30608354	A/G	3‘-UTR
*SNAP29*	Synaptosomal-associated protein 29	26:30608375	C/T	3‘-UTR
*SNAP29*	Synaptosomal-associated protein 29	26:30608864	A/G	3‘-UTR
*SNAP29*	Synaptosomal-associated protein 29	26:30608989	C/T	3‘-UTR
*SNAP29*	Synaptosomal-associated protein 29	26:37750228	T/A	3‘-UTR
*SNAP29*	Synaptosomal-associated protein 29	26:38554416	G/A	3‘-UTR
*STX7*	Syntaxin-7	1:25559797	T/C	3‘-UTR
*TH*	Tyrosine 3-monooxygenase	18:46331581	G/A	Splice region, intronic

Strikingly, polymorphisms in three of these genes have previously been associated with aggressive behaviour within (*SLC6A3* [24]) or between (*TH* [25], *MAOB* [22]) dog breeds. However the alleles in these studies differ from those that we identify. This suggests that the catecholamine pathway has been recurrently targeted by selection during the process of dog domestication. Furthermore, some genes in this pathway show evidence of being recurrently selected during the process of dog domestication, with some variants contributing to behavioral differences between dogs and wolves and others to differences between dog breeds.

We note that a previous study by Li et al. [35] identified genes involved in glutamate metabolism as the most highly diverged between dogs and wolves. We do not detect this signal in our analysis. This may be partially due to the larger sample size in our study (78 compared to 13 canid genomes), which gives us greater power to detect variants that are truly highly diverged between dogs and wolves. Another explanation is that the analysis of Li et al. [35] was designed to identify genes with highly divergent SNPs irrespective of whether they contain putatively functional variants. Therefore, there may indeed be selection on glutamate metabolism genes in dogs, but the selected variants may reside in nearby regulatory elements. This is supported by their finding that there are gene expression changes in these genes between dogs and wolves [35].

In contrast, our analysis was designed to identify genes with highly divergent putatively functional variants within, or neighbouring, exonic sequences. Therefore, the differing results could be due to selection on the ‘adrenaline and noradrenaline biosynthesis pathway’ occurring via modifications to the protein structure (missense mutations in *DDC* and *SNAP29*) and flanking proximal regulatory regions (5′-UTR, 3′-UTR and intronic splice sites) of selected genes. While selection on glutamate metabolism may have primarily occurred via selection on more distal regulatory elements, such as enhancers, potentially influencing tissue specific gene expression. Given the highly polygenic nature of domestication [70], it is plausible that both these pathways have been targeted by selection during dog domestication.

### Characterizing the frequency distribution of putatively selected variants

**Fig. 2:**
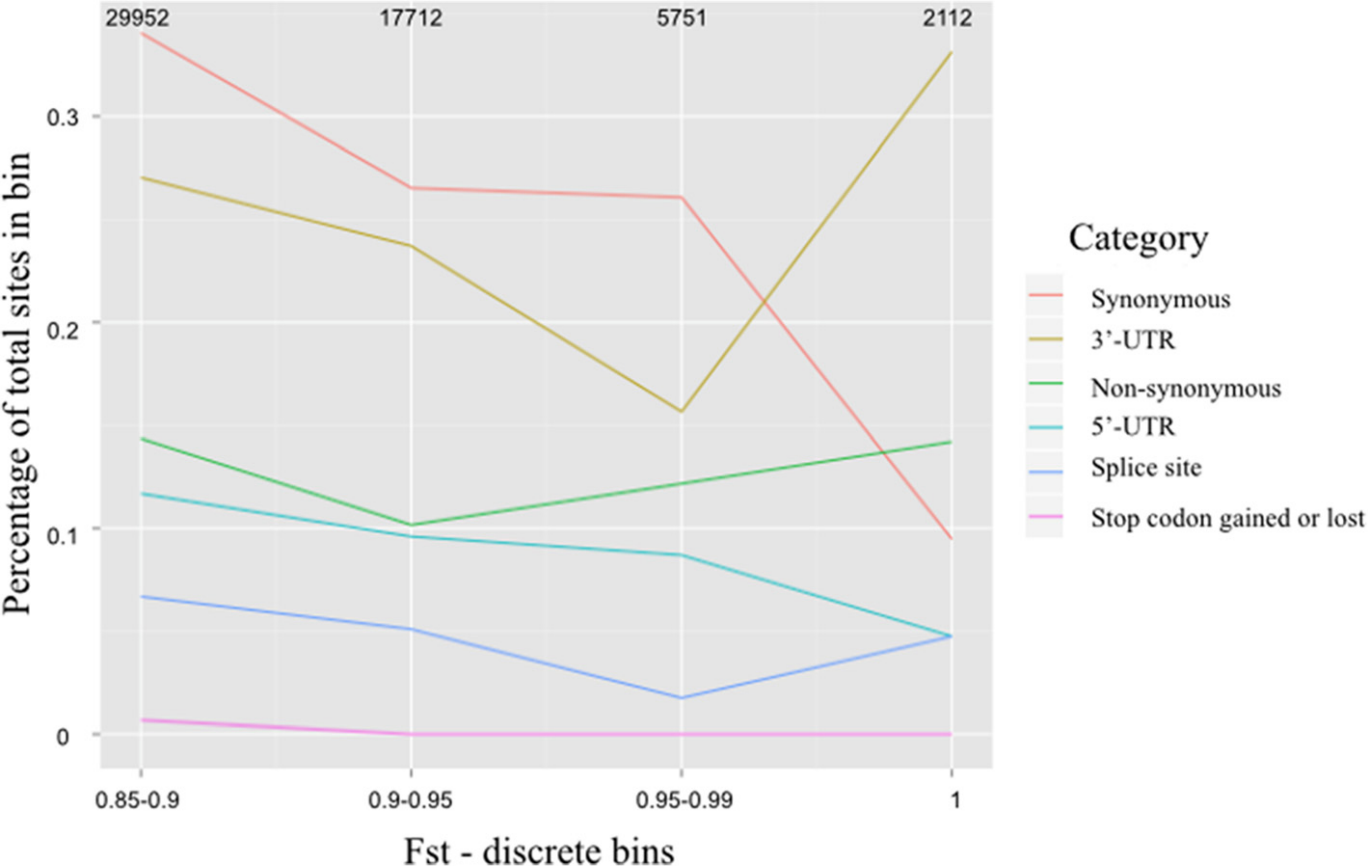
Percentage of functional sites in discrete Fst bins. Polymorphic sites and substitutions were ordered into bins based on their Fst score. For each bin sites were categorized according to their putative function. The number of sites in each functional category are plotted as a percentage of the total sites in that bin. Values at top refer to total number of sites in each bin. Synonymous sites, which are assumed to be selectively neutral, decline as a percentage of total sites as the Fst score of the bin increases. In contrast, we observe an increase in the percentage of several categories functional sites in the top bins [0.95–1, 1]. This may be the result of positive selection raising these variants to high frequency and fixation

It has been proposed that animal domestication is highly polygenic and can be achieved by the concordant increase in allele frequency of multiple variants without fixation at any loci [70]. We ordered putatively selected sites into bins based on their Fst score [0.85–0.9, 0.9–0.95, 0.95–1]. For each discrete bin sites were further categorized based on their putative functional consequences using VEP. The percentage of sites in each functional category are plotted for each bin as a percentage of total sites in that bin (Fig. 2). In the absence of positive selection we expect the proportion of putatively functional variants to decrease as Fst increases because purifying selection should act to prevent deleterious mutations rising in frequency [71]. Indeed, for Fst values between 0.85-0.95 we see the proportion of all categories of putatively functional sites decreasing as Fst increases (Fig. 2). However, for Fst values >0.95 we see an increase in the percentage of several categories of putatively functional sites, particularly sites in the 3′-UTR of genes, while the percentage of synonymous sites, which are presumed to be selectively neutral, decreases. This is suggestive of positive selection acting to bring these variants to fixation.

### Evidence that the strength of selection varies around different categories of sites

**Fig. 3:**
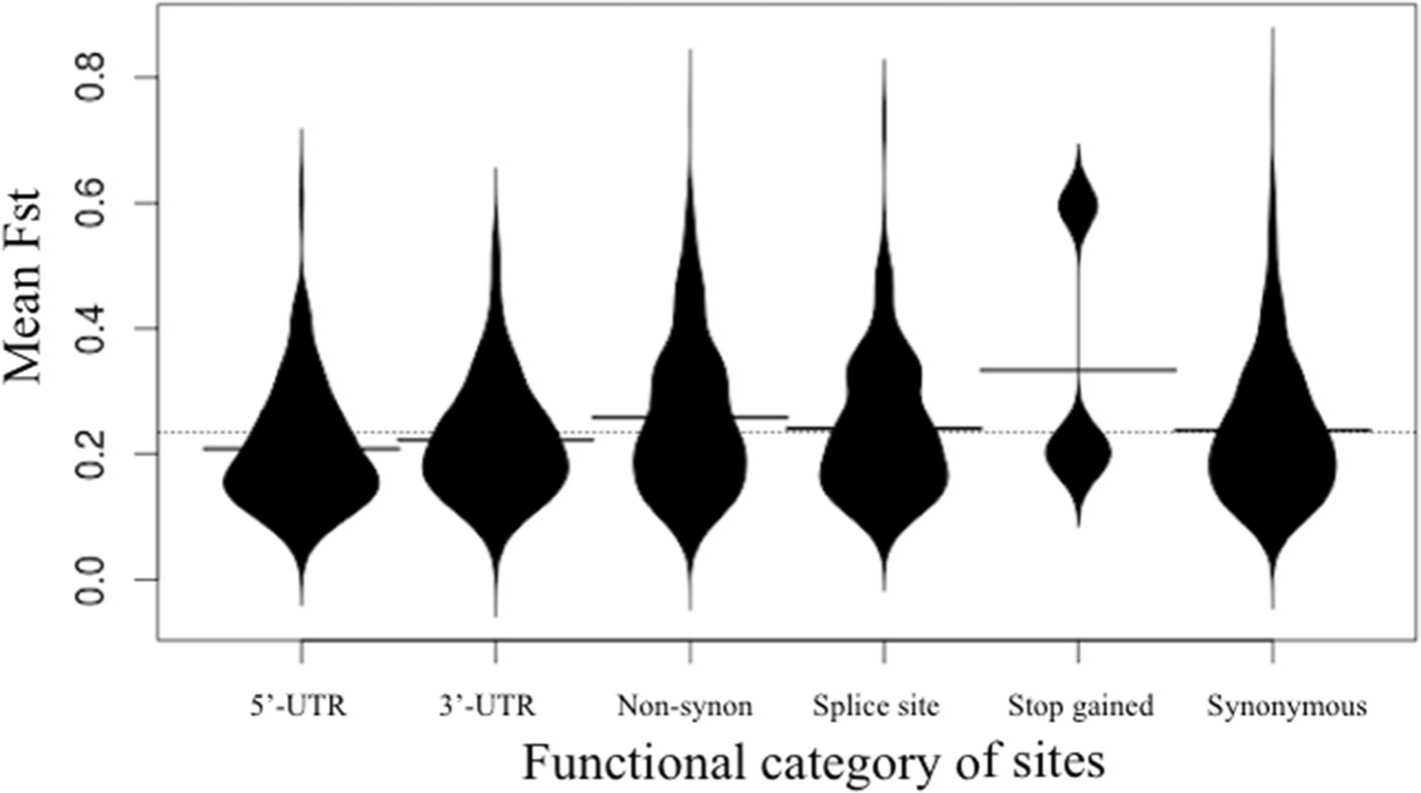
Distribution of mean Fst in 50kb windows centered around putatively functional sites. Polymorphic sites with Fst > =0.75 between dogs and wolves were classified according to their putative function. For each putatively functional site the mean Fst was calculated in a 50kb window centered on the site. A violin plot shows the distribution of mean Fst values for each category of functional site (5‘-UTR, 3‘-UTR, non-synonymous, splice site, stop gained, synonymous). Synonymous mutations were included as a category to show the expectation in the absence of positive selection

To further investigate whether selection has preferentially acted on any specific functional categories of sites we calculated mean Fst in 50kb windows centered on each putatively functional variant with an Fst score > = 0.75. Figure 3 shows the distribution of mean Fst around the difference categories of sites, with synonymous variants acting as a control as we do not expect positive selection to be acting on synonymous sites, although this assumption may not always be valid [72]. An ANOVA reveals a significant effect of functional category on mean Fst around sites, F[5, 2818] = 10.98, *p* = 1.71e-10 (Additional file 2: Table S1). To find which categories are significantly different we performed Tukey’s range test. Although mean Fst is highest around sites that cause a gain of stop codon this is not significantly different as there are only three such sites. We find that non-synonymous variants are in regions of significantly elevated Fst compared to synonymous variants, an observation consistent with positive selection acting on non-synonymous sites (Additional file 3: Table S2). Interestingly, both synonymous and non-synonymous variants appear to be in regions of significantly higher Fst than variants in the 3′-UTRS and 5′-UTRs. This suggests that during dog domestication selection may have been strongest around non-synonymous variants. However, there are more non-coding than coding variants segregating at high frequency between dogs and wolves, so the overall contribution of each type of variant may still be similar. The elevated mean Fst around synonymous sites relative to regulatory variants may be the result of hitchhiking of synonymous sites that are on the same haplotype as selected variants or, less plausibly, selection on synonymous sites.

## Conclusions

Using genome-wide polymorphism data from dogs and wolves we were able to identify putatively functional variants that may have been selected during dog domestication. While previous genomic studies of dog domestication have identified putatively selected regions and genes, this is the first study to combine scans for selection with a genome-wide analysis of multiple categories of putatively functional variants in order to identify specific genetic changes underlying the phenotypic differences between dogs and wolves. We find there are only 11 genes with putatively functional substitutions differentiating all dogs and wolves. Although we note this is likely to be an under-estimate due to our currently limited ability to identify functional variation in non-genic regions of the genome. The 11 genes that we detect with fixed functional differences between dogs and wolves point towards selection on both morphological and behavioral phenotypes.

We find that, although the majority of putatively functional variants segregating between dogs and wolves are in regulatory regions, in general variants influencing protein structure show the strongest signatures of selection. Although we note that our analysis was restricted to regulatory regions in close proximity to genes. In the future, characterizing the functional effects of these variants may help to further our understanding of the domestication process.

The majority of variants that we detect segregating between dogs and wolves are not fixed but may nevertheless contribute to differences between dogs and wolves due to the polygenic nature of most phenotypes. We provide the first evidence for polygenic selection on putatively functional variation in genes in the adrenaline and noradrenaline biosynthesis pathway during dog domestication. The genes we find implicated in this pathway are involved in the synthesis, transport and degradation of a variety of neurotransmitters, particularly the catecholamines, which include dopamine and noradrenaline. The strong signal of recurrent selection on this pathway and its role in emotional processing and the fight-or-flight response suggests that the behavioral changes we see in dogs compared to wolves may in part be due to changes in this pathway. Furthermore, several of the genes contributing to the signal of enrichment in this pathway have also been associated with levels of aggressive behaviour between dog breeds [22, 25], suggesting that some of these genes have been important during both the initial domestication process and later breed formation. We note that although the high allele frequency differences between dogs and wolves suggest that the variants we identify were involved in the early domestication process, it is possible that the allelic differentiation we observe occurred later. Looking ahead, ancient DNA from dogs and wolves may provide the temporal resolution to determine which alleles were involved in the earliest stages of dog domestication.

## Methods

### Data & samples

We used the DoGSD, a publicly available database which contains whole-genome SNP data from dogs and wolves conglomerated from several different studies [37]. All data were obtained from this database and no animal experiments were conducted. For comparability between datasets DoGSD applies a unified variant calling pipeline to all the samples. Using this dataset we analyzed whole-genome variant data from 67 dog and 7 wolf samples (Additional file 4: Table S3), which we treated as two separate groups. The strong genetic drift caused by breed specific population bottlenecks associated with breed creation has resulted in the random fixation of large genomic regions [73]. These could be misidentified as signals of selection. However, we are interested in variants that were selected for during the early domestication process, before the creation of modern breeds. By combining data from as many dogs as possible, from both modern breeds and village dog populations, we hope to alleviate this problem. Basing our analysis on the reasonable assumption that dog domestication had a single origin [1], we expect variants that were strongly selected for during the early domestication process to be shared across dog breeds, regardless of their more recent population history. While the neutral regions that underwent fixation during breed formation are not expected to be shared across all breeds due to the random nature of genetic drift. Although we note that some variants that were selected for during the early domestication process could be absent from some breeds due to drift from strong bottlenecks associated with the breed creation process.

We excluded the dingo (*Canis lupus dingo*) because although they are now wild, they are thought to be descended from a domesticated Asian dog population [74], which could lead to false negative results if they still contain alleles that were selected for during the early domestication process. To visualize the relationship between samples we created a PCA plot of the samples included in all analyses using EIGENSOFT and SMARTPCA [75, 76] (Additional file 5: Figure S1). The first principal component in the PCA plot clearly differentiates wolves and domestic dogs into two groups. The second principal component appears to differentiate dogs based on their Asian and European ancestry. To reduce the potential for false positives due to low power we only considered sites with genotype calls for > = 50 % of samples among both the dogs and the wolves.

### Genomic scan for selection

To identify regions of the genome with putative signatures of positive selection in dogs or wolves we calculated mean Fst across the genome between dogs and wolves in non-overlapping 500kb windows using VCFtools [77]. This is an implementation of Weir and Cockerham’s Fst [78]. Under neutrality we expect the distribution of mean Fst scores to follow a normal distribution. However a histogram of mean Fst scores shows a long tail towards positive Fst scores, potentially indicative of positive selection (Additional file 6: Figure S2).

### Pathway enrichment analysis

Pathway enrichment analysis was performed using the gene ontology and analysis software PANTHER [59 60]. We performed the statistical overrepresentation test using the *Canis familiaris* background gene set and applied the bonferroni correction for multiple hypothesis testing.

### Identification of putatively functional sites

The majority of genomic variants are expected to have no impact on the phenotype of an organism. To identify the putatively functional sites that may have been targeted by selection we used Ensembl’s Variant Effect Predictor [VEP] [41]. The VEP predicts the effect of genomic variants on genes, protein sequence and regulatory regions. We classify as putatively functional any sites that influence protein structure; cause missense mutations, frameshifts, or gain or loss of stop codons, and variants that may influence gene expression by being within a 5′-UTR, 3′-UTR, or predicted splice site. While this categorization is likely to be overly conservative, by excluding potentially regulatory variants not situated in or near genes, it will reduce the number of false positives by only including variants with a high probability of having functional consequences.

### Coalescent simulations

To test whether the putatively selected 500kb windows with elevated mean Fst between dogs and wolves could be the result of a selectively neutral demographic history we performed coalescent simulations with the software scrm [79]. The parameters for the simulations were taken from the papers where the samples were first presented. Specifically, we adapted the demographic model presented in [1] (Supplementary Text 8, Command Line 1 *G-PhoCS* model with the full set of migration bands inferred) and incorporated demographic information from the papers where the additional samples were presented [34, 80]. We simulated 148 500kb haplotypes 6000 times, to provide a distribution of regions approximating the dog genome in size. The exact command line is presented in Additional file 7: Table S4. For each simulation we calculated the mean Fst of the 500kb haplotypes between dogs and wolves using the R package PopGenome [81].

## Availability of supporting data

The dataset supporting the conclusions of this article is available in the DoGSD repository [37] [http://dogsd.big.ac.cn/snp/pages/download/download.jsp].

## Additional files

Additional file 1: Figure S3. Mean Fst of 500kb regions. Distribution of the empirical data compared to results obtained from coalescent simulations. The empirical distribution is presented both with (red line) and without the regions from the X chromosome (blue line). The long tail of the empirical data is absent in the neutral simulations, suggesting that positive selection may explain the elevated Fst in these regions. (DOCX 73 kb) Additional file 2: Table S1. Results of ANOVA of mean Fst in 50kb windows around functional categories of sites with Fst > = 0.75. (DOCX 41 kb) Additional file 3: Table S2. Results of Tukey’s range test for ANOVA of Mean Fst in 50kb windows around functional categories of sites with Fst > = 0.75. (DOCX 99 kb) Additional file 4: Table S3. Samples from the DoGSD included in this study. (DOCX 121 kb) Additional file 5: Figure S1. PCA plot of samples included in this study. PCA of genome-wide polymorphism data from 67 dogs and 7 wolves. The percentage of the total variance explained by the first and second principal component are labeled on the X and Y axis, respectively. PC1 clearly separates dogs from wolves while PC2 primarily separates dogs by geographic origin. (DOCX 144 kb) Additional file 6: Figure S2. Histogram of mean Fst scores calculated in 500kb windows genome-wide between dogs and wolves. Histogram of mean Fst calculated in 500kb genomic windows across the autosome and X chromosome between dogs and wolves. Counts are included above each bin. The long tail towards positive mean Fst scores is potentially indicative of positive selection. (DOCX 71 kb) Additional file 7: Table S4. The scrm command line used for coalescent simulations of dog and wolf demographic history and Ne estimates and parameters used for the simulations. (DOCX 77 kb)
