# Supplementary material for: Identification of genomic variants putatively targeted by selection during dog domestication
Source: BMC Evol Biol. 2016 Jan 12;16:10. doi: 10.1186/s12862-015-0579-7 (PMC4710014; doi:10.1186/s12862-015-0579-7)

**Supplementary Figure 3. Mean Fst of 500kb regions. Distribution of the empirical data compared to results obtained from coalescent simulations.**


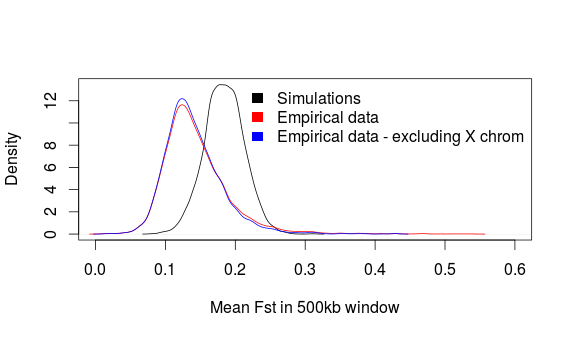

Supplement: Additional file 1: Figure S3. — Mean Fst of 500kb regions. Distribution of the empirical data compared to results obtained from coalescent simulations. The empirical distribution is presented both with (red line) and without the regions from the X chromosome (blue line). The long tail of the empirical data is absent in the neutral simulations, suggesting that positive selection may explain the elevated Fst in these regions. (DOCX 73 kb) [file 12862_2015_579_MOESM1_ESM.docx]
