# Supplementary material for: Identification of genomic variants putatively targeted by selection during dog domestication
Source: BMC Evol Biol. 2016 Jan 12;16:10. doi: 10.1186/s12862-015-0579-7 (PMC4710014; doi:10.1186/s12862-015-0579-7)
Supplement: Additional file 2: Table S1. — Results of ANOVA of mean Fst in 50kb windows around functional categories of sites with Fst > = 0.75. (DOCX 41 kb) [file 12862_2015_579_MOESM2_ESM.docx]

**Supplementary Table 1. Results of ANOVA of mean Fst in 50kb windows around functional categories of sites with Fst >= 0.75**

|  | Df | Sum Sq | Mean Sq | F value | Pr(>F) |
| --- | --- | --- | --- | --- | --- |
| Functional categories | 5 | 0.63 | 0.12544 | 10.98 | 1.71e-10 |
| Residuals | 2818 | 32.18 | 0.01142 |  |  |
