# Supplementary material for: Identification of genomic variants putatively targeted by selection during dog domestication
Source: BMC Evol Biol. 2016 Jan 12;16:10. doi: 10.1186/s12862-015-0579-7 (PMC4710014; doi:10.1186/s12862-015-0579-7)
Supplement: Additional file 3: Table S2. — Results of Tukey’s range test for ANOVA of Mean Fst in 50kb windows around functional categories of sites with Fst > = 0.75. (DOCX 99 kb) [file 12862_2015_579_MOESM3_ESM.docx]

**Supplementary Table 2. Results of Tukey's range test for ANOVA of Mean Fst in 50kb windows around functional categories of sites with Fst >= 0.75**

| Comparison | Difference in observed means | Lower interval | Upper interval | Adjusted P-value |
| --- | --- | --- | --- | --- |
| 5’-UTR-3’-UTR | -0.014384125 | -0.037221200 | 0.008452951 | 0.4683529 |
| Non_synonymous-3’-UTR | 0.035246115 | 0.018772050 | 0.051720181 | 0.0000000 |
| Splice_site-3’-UTR | 0.018374072 | -0.007359839 | 0.044107983 | 0.3220012 |
| Stop_gained-3’-UTR | 0.110713406 | -0.065507770 | 0.286934581 | 0.4713171 |
| Synonymous-3’-UTR | 0.015509507 | 0.001511038 | 0.029507977 | 0.0198602 |
| Non_synonymous-5’-UTR | 0.049630240 | 0.025256569 | 0.074003911 | 0.0000001 |
| Splice_site-5’-UTR | 0.032758197 | 0.001374838 | 0.064141555 | 0.0348514 |
| Stop_gained-5’-UTR | 0.125097530 | -0.052036836 | 0.302231896 | 0.3344823 |
| Synonymous-5’-UTR | 0.029893632 | 0.007120004 | 0.052667260 | 0.0025500 |
| Splice_site-Non_synonymous | -0.016872043 | -0.043978832 | 0.010234745 | 0.4822043 |
| Stop_gained-Non_synonymous | 0.075467290 | -0.100959597 | 0.251894177 | 0.8273125 |
| Synonymous-Non_synonymous | -0.019736608 | -0.036122607 | -0.003350609 | 0.0079127 |
| Stop_gained-Splice_site | 0.092339334 | -0.085191750 | 0.269870418 | 0.6751021 |
| Synonymous-Splice_site | -0.002864565 | -0.028542187 | 0.022813058 | 0.9995681 |
| Synonymous-Stop_gained | -0.095203898 | -0.271416863 | 0.081009066 | 0.6378315 |
