# Supplementary material for: Identification of genomic variants putatively targeted by selection during dog domestication
Source: BMC Evol Biol. 2016 Jan 12;16:10. doi: 10.1186/s12862-015-0579-7 (PMC4710014; doi:10.1186/s12862-015-0579-7)
Supplement: Additional file 4: Table S3. — Samples from the DoGSD included in this study. (DOCX 121 kb) [file 12862_2015_579_MOESM4_ESM.docx]

**Supplementary Table 3. Samples from the DoGSD included in this study**

| **ID** | **Breed** | **Sample Location** | **Coverage(X)** |
| --- | --- | --- | --- |
| GS1 | German Shepherd | Kunming | 15.66 |
| GS2 | German Shepherd | Kunming | 16.44 |
| GS3 | German Shepherd | Kunming | 16.07 |
| GS4 | German Shepherd | Kunming | 15.97 |
| GS5 | German Shepherd | Kunming | 15.24 |
| GS6 | German Shepherd | Kunming | 16.27 |
| GS7 | German Shepherd | Kunming | 16.86 |
| GS8 | German Shepherd | Kunming | 15.33 |
| GS9 | German Shepherd | Kunming | 16.93 |
| GS10 | German Shepherd | Kunming | 15.98 |
| KM1 | Indigenous dog | Kunming | 12.01 |
| KM2 | Indigenous dog | Kunming | 17.32 |
| KM3 | Indigenous dog | Kunming | 16.48 |
| KM4 | Indigenous dog | Kunming | 16.92 |
| KM5 | Indigenous dog | Kunming | 16.73 |
| KM6 | Indigenous dog | Kunming | 16.23 |
| KM7 | Indigenous dog | Kunming | 18.14 |
| KM8 | Indigenous dog | Kunming | 14.44 |
| KM9 | Indigenous dog | Kunming | 14.74 |
| KM10 | Indigenous dog | Kunming | 17.21 |
| YJ1 | Indigenous dog | Yingjiang | 16.81 |
| YJ2 | Indigenous dog | Yingjiang | 15.03 |
| YJ3 | Indigenous dog | Yingjiang | 14.71 |
| YJ4 | Indigenous dog | Yingjiang | 14.94 |
| YJ5 | Indigenous dog | Yingjiang | 14.42 |
| YJ6 | Indigenous dog | Yingjiang | 15.32 |
| YJ7 | Indigenous dog | Yingjiang | 15.17 |
| YJ8 | Indigenous dog | Yingjiang | 15.54 |
| YJ9 | Indigenous dog | Yingjiang | 15.55 |
| YJ10 | Indigenous dog | Yingjiang | 16.22 |
| LJ1 | Indigenous dog | Lijiang | 16.76 |
| LJ2 | Indigenous dog | Lijiang | 16.72 |
| LJ3 | Indigenous dog | Lijiang | 15.97 |
| LJ4 | Indigenous dog | Lijiang | 14.35 |
| LJ5 | Indigenous dog | Lijiang | 15.28 |
| LJ6 | Indigenous dog | Lijiang | 15.78 |
| LJ7 | Indigenous dog | Lijiang | 13.54 |
| LJ8 | Indigenous dog | Lijiang | 16.03 |
| LJ9 | Indigenous dog | Lijiang | 16.02 |
| LJ10 | Indigenous dog | Lijiang | 15.38 |
| DQ1 | Indigenous dog | Diqing | 16.60 |
| DQ2 | Indigenous dog | Diqing | 17.23 |
| DQ3 | Indigenous dog | Diqing | 15.13 |
| DQ4 | Indigenous dog | Diqing | 15.81 |
| DQ5 | Indigenous dog | Diqing | 16.48 |
| DQ6 | Indigenous dog | Diqing | 13.85 |
| DQ7 | Indigenous dog | Diqing | 17.00 |
| DQ8 | Indigenous dog | Diqing | 13.27 |
| DQ9 | Indigenous dog | Diqing | 14.97 |
| DQ10 | Indigenous dog | Diqing | 16.37 |
| TM1 | Tibetan Mastiff | Diqing | 16.07 |
| TM2 | Tibetan Mastiff | Diqing | 14.34 |
| TM3 | Tibetan Mastiff | Diqing | 14.66 |
| TM4 | Tibetan Mastiff | Diqing | 13.91 |
| TM5 | Tibetan Mastiff | Diqing | 14.91 |
| TM6 | Tibetan Mastiff | Diqing | 15.51 |
| TM7 | Tibetan Mastiff | Diqing | 16.63 |
| TM8 | Tibetan Mastiff | Diqing | 14.74 |
| TM9 | Tibetan Mastiff | Diqing | 16.71 |
| TM10 | Tibetan Mastiff | Diqing | 16.70 |
| FAMICHN00001 | Indigenous dog | Xi'an, China | 19.10 |
| FAMICHN00002 | Indigenous dog | Simao, China | 11.43 |
| FAMICHN00003 | Indigenous dog | Ya'an, China | 12.63 |
| LUPWRUS00001 | Grey wolf | Altai, Russia | 11.32 |
| LUPWRUS00002 | Grey wolf | Chukotka, Russia | 11.59 |
| LUPWRUS00003 | Grey wolf | Bryansk, Russia | 30.73 |
| LUPWCHN00001 | Grey wolf | Inner Mongolia, China | 19.05 |
| FAMBGSD00001 | German Shepherd Dog | NA | 9.53 |
| FAMBTIM00001 | Tibetan Mastiff | NA | 10.99 |
| FAMBBEM00001 | Belgian Malinois | NA | 9.97 |
| Basenji | Basenji | Bethesda, MD, USA | 4.25 |
| CHW | Wolf | San Diego Zoo, CA, USA | 19.62 |
| CRW | Wolf | Perković, Croatia | 6.70 |
| ISW | Wolf | Neve Ativ, Golan Heights, Israel | 5.04 |
