# Supplementary figures and images for: Identification of genomic variants putatively targeted by selection during dog domestication
Source: BMC Evol Biol. 2016 Jan 12;16:10. doi: 10.1186/s12862-015-0579-7 (PMC4710014; doi:10.1186/s12862-015-0579-7)

**Supplementary Figure 1**

**
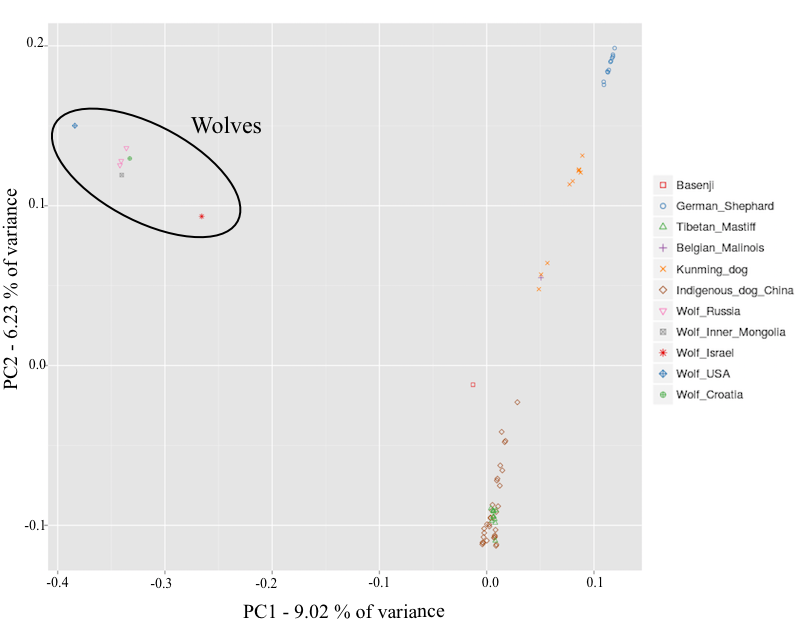
**

Supplement: Additional file 5: Figure S1. — PCA plot of samples included in this study. PCA of genome-wide polymorphism data from 67 dogs and 7 wolves. The percentage of the total variance explained by the first and second principal component are labeled on the X and Y axis, respectively. PC1 clearly separates dogs from wolves while PC2 primarily separates dogs by geographic origin. (DOCX 144 kb) [file 12862_2015_579_MOESM5_ESM.docx]

**Supplementary Figure 2**

**
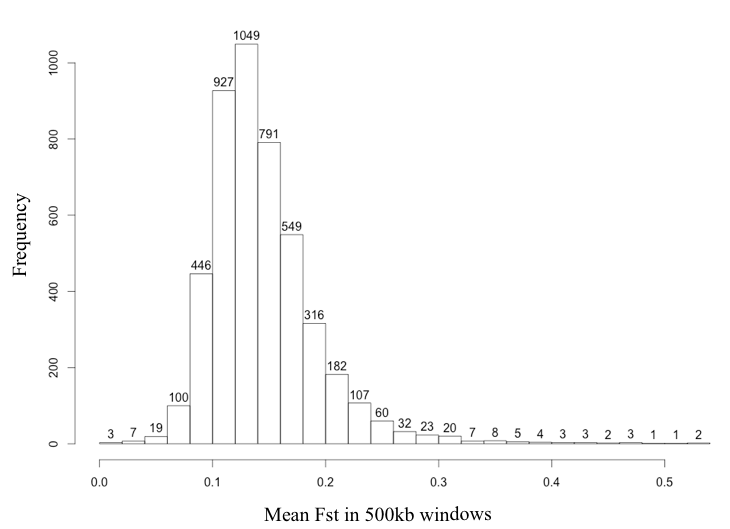
**

Supplement: Additional file 6: Figure S2. — Histogram of mean Fst scores calculated in 500kb windows genome-wide between dogs and wolves. Histogram of mean Fst calculated in 500kb genomic windows across the autosome and X chromosome between dogs and wolves. Counts are included above each bin. The long tail towards positive mean Fst scores is potentially indicative of positive selection. (DOCX 71 kb) [file 12862_2015_579_MOESM6_ESM.docx]
