# Supplementary material for: Identification of genomic variants putatively targeted by selection during dog domestication
Source: BMC Evol Biol. 2016 Jan 12;16:10. doi: 10.1186/s12862-015-0579-7 (PMC4710014; doi:10.1186/s12862-015-0579-7)
Supplement: Additional file 7: Table S4. — The scrm command line used for coalescent simulations of dog and wolf demographic history and Ne estimates and parameters used for the simulations. (DOCX 77 kb) [file 12862_2015_579_MOESM7_ESM.docx]

**Supplementary Table 4**. **The scrm command line used for coalescent simulations of dog and wolf demographic history and Ne estimates and parameters used for the simulations.**

scrm 148 6000 -t 900 -r 225 500000 -I 9 6 4 2 2 86 22 22 2 2 -n 1 0.12 -n 2 0.12 -n 3 0.253 -n 4 0.581 -n 5 0.378 -n 6 0.0036 -n 7 0.0036 -n 8 0.0036 -n 9 0.0578 -m 4 9 13500 -m 9 4 32400 -m 2 5 2358 -m 1 5 2358 -m 5 2 3078 -m 5 1 3078 -ej 0.0248 2 1 -ej 0.0248 3 1 -ej 0.0248 4 1 -en 0.0248 1 0.2889 -ej 0.000213 8 7 -ej 0.009259 7 6 -ej 0.022407 6 5 -ej 0.022407 9 5 -ej 0.027593 5 1 -em 0.022407 9 4 0 -em 0.022407 4 9 0 -em 0.022407 5 2 0 -em 0.022407 2 5 0 -em 0.022407 1 5 0 -em 0.022407 5 1 0 -en 0.0248 1 0.2889 -en 0.022407 5 0.0433 -en 0.027593 1 1

| **Population** | **Number of haplotypes** | **Present day Ne** |
| --- | --- | --- |
| Russian grey wolf | 6 | 5400 |
| Chinese grey wolf | 4 | 5400 |
| Croatian grey wolf | 2 | 11400 |
| Israeli grey wolf | 2 | 26150 |
| Indigenous dog | 86 | 17000 |
| Tibetan mastiff | 22 | 200 |
| German shephard | 22 | 200 |
| Belgian malinois | 2 | 200 |
| Basenji | 2 | 2600 |
|  |  |  |
| Mutation rate | 1x10E-8 mutations per generation |  |
| Recombination rate | 0.25cM/Mb |  |
| Generation time | 3 years |  |
